# Genetic Variants on 3q21 and in the Sp8 Transcription Factor Gene (*SP8*) as Susceptibility Loci for Psychotic Disorders: A Genetic Association Study

**DOI:** 10.1371/journal.pone.0070964

**Published:** 2013-08-13

**Authors:** Kenji Kondo, Masashi Ikeda, Yusuke Kajio, Takeo Saito, Yoshimi Iwayama, Branko Aleksic, Kazuo Yamada, Tomoko Toyota, Eiji Hattori, Hiroshi Ujike, Toshiya Inada, Hiroshi Kunugi, Tadafumi Kato, Takeo Yoshikawa, Norio Ozaki, Nakao Iwata

**Affiliations:** 1 Department of Psychiatry, Fujita Health University School of Medicine, Toyoake, Aichi, Japan; 2 Laboratory for Molecular Psychiatry, RIKEN Brain Science Institute, Wako, Saitama, Japan; 3 Department of Psychiatry, Nagoya University Graduate School of Medicine, Nagoya, Aichi, Japan; 4 Department of Neuropsychiatry, Okayama University Graduate School of Medicine, Dentistry and Pharmaceutical Science, Okayama, Okayama, Japan; 5 Department of Psychiatry, Seiwa Hospital, Institute of Neuropsychiatry, Shinjuku, Tokyo, Japan; 6 Department of Mental Disorder Research, National Institute of Neuroscience, National Center of Neurology and Psychiatry, Kodaira, Tokyo, Japan; 7 Laboratory for Molecular Dynamics of Mental Disorders, RIKEN Brain Science Institute, Wako, Saitama, Japan; United Graduate School of Child Development, Osaka University, Japan

## Abstract

**Background:**

Recent genome-wide association studies (GWASs) investigating bipolar disorder (BD) have detected a number of susceptibility genes. These studies have also provided novel insight into shared genetic components between BD and schizophrenia (SCZ), two major psychotic disorders. To examine the replication of the risk variants for BD and the pleiotropic effect of the variants associated with BD, we conducted a genetic association study of single nucleotide polymorphisms (SNPs) that were selected based upon previous BD GWASs, which targeted psychotic disorders (BD and SCZ) in the Japanese population.

**Methods:**

Forty-eight SNPs were selected based upon previous GWASs. A two-stage analysis was conducted using first-set screening (for all SNPs: BD = 1,012, SCZ = 1,032 and control = 993) and second-set replication samples (for significant SNPs in the screening analysis: BD = 821, SCZ = 1,808 and control = 2,149). We assessed allelic association between BD, SCZ, psychosis (BD+SCZ) and the SNPs selected for the analysis.

**Results:**

Eight SNPs revealed nominal association signals for all comparisons (P_uncorrected_<0.05). Among these SNPs, the top two SNPs (associated with psychosis: P_corrected_ = 0.048 and 0.037 for rs2251219 and rs2709722, respectively) were further assessed in the second-set samples, and we replicated the signals from the initial screening analysis (associated with psychosis: P_corrected_ = 0.0070 and 0.033 for rs2251219 and rs2709722, respectively). The meta-analysis between the current and previous GWAS results showed that rs2251219 in Polybromo1 (*PBRM1*) was significant on genome-wide association level (P = 5×10^−8^) only for BD (P = 9.4×10^−9^) and psychosis (P = 2.0×10^−10^). Although the association of rs2709722 in Sp8 transcription factor (*SP8*) was suggestive in the Asian population (P = 2.1×10^−7^ for psychosis), this signal weakened when the samples size was increased by including data from a Caucasian population (P = 4.3×10^−3^).

**Conclusions:**

We found 3p21.1 (including *PBRM1*, strong linkage disequilibrium made it difficult to pinpoint the risk genes) and *SP8* as risk loci for BD, SCZ and psychosis. Further replication studies will be required for conclusive results.

## Introduction

Bipolar disorder (BD) is a severe mental condition, and the main symptom is associated with abnormal affective status (i.e., a patient's mood will swing from manic to depression or *vice-versa)*. The prevalence of BD worldwide is greater than 1% [1], [2], but the precise molecular mechanism is largely unknown. Nevertheless, epidemiological surveys have suggested that genetic factors contribute substantially compared with the environmental factors, and heritability has been estimated at 80% [3].

The results of genetic association studies, particularly genome-wide association studies (GWASs), have identified an increasing number of risk genes for susceptibility to BD. The initial meta-analysis of the GWASs detected a single nucleotide polymorphism (SNP) in the α subunit of the L-type voltage-gated calcium channel (*CACNA1C*) with suggestive statistical evidence for association (P = 7×10^−8^) [4]. This gene remains one of the most promising genes for BD, as the most recent mega-analysis in the Psychiatric GWAS Consortium (PGC) Bipolar Working Group provided stronger evidence of association with the SNPs in *CACNA1C* (P = 1.5×10^−8^) [5]. This mega-analysis also detected another risk SNP in Drosophila pair-rule gene ten-m (*ODZ4*) with genome-wide significance (P = 4.4×10^−8^), proposing a novel candidate gene for BD [5]. Other studies based upon genome-wide screening methodology identified several possible candidate genes for BD, such as ankyrin 3 (*ANK3*) [4] and neurocan (*NCAN*) [6].

Interestingly, genetic association studies of schizophrenia (SCZ), which is another major psychotic disorder, have also revealed that the BD risk SNPs, such as *CACNA1C* and *ANK3*, are associated with SCZ [7]. This result indicates that there is a shared genetic component between BD and SCZ. Furthermore, several independent lines of evidence converge to support this hypothesis: first, polygenic component analysis, in which the cumulative number of liberal risk alleles for SCZ or BD were enriched in the patients with BD or SCZ [8], and second, epidemiological studies, in which relatives of a proband with BD also had elevated risk for SCZ in addition to BD [9]. Therefore, combining BD and SCZ samples into a single psychosis group [10] can provide increased statistical power, specifically for the detection of overlapping risk SNPs. Several studies have used this concept also enhance detection of risk SNPs for either BD or SCZ [11], [12].

The goal of the current study was to conduct replication and cross-phenotype analyses of the SNPs that were selected based upon BD GWAS findings with two psychotic disorders (BD and SCZ) in the Japanese population. We used a two-stage design (screening and replication samples) and a meta-analysis approach to obtain reliable results.

## Materials and Methods

### Ethics Statement

Written informed consent was obtained from each subject after the procedures had been fully explained. This study was performed in accordance with the World Medical Association's Declaration of Helsinki and approved by the ethics committees at Fujita Health University, RIKEN BSI and institutes participating in the Collaborative Study of Mood Disorder (COSMO) [13].

### Subjects

We used two independent sample sets in this study. In the first-set screening analysis, we examined 1,012 patients with BD [51.8% female, age ± standard deviation (SD) = 50.7 ± 14.3 years, BD type I = 621, BD type II = 380, schizoaffective disorder (SA) = 7, BD type information not available = 4], 1,032 SCZ (48.3% female, mean ± SD = 46.8 ± 14.8 years) and 993 healthy controls (51.1% female, age ± SD = 49.7 ± 14.0 years).

For the two SNPs that showed a significant association in the screening analysis, we used an independent second-set of samples for replication analysis. This sample consisted of 821 patients with BD (54.6% female, age ± SD = 48.2±14.4, BD type I = 387, BD type II = 344, SA = 89, BD type information not available = 1), 1,808 patients with SCZ (45.1% female, age ± SD = 49.8±14.8 years) and 2,149 healthy controls (58.3% female, age ± SD = 42.3±14.2 years). A detailed description of our subjects, including a general characterization and psychiatric assessment, is described elsewhere [13].

### SNP selection and quality control

We selected 48 SNPs from BD GWAS data published prior to September 2011 [4], [5], [6], [14], [15], [16], [17], [18], [19], [20]. Regarding SNP selection, we used the following inclusion criteria. The potential risk SNPs in autosomal chromosomes must have had a P-value less than 1×10^−5^ if the original GWAS was conducted using a Caucasian population. The P-value must have been less than 1×10^−4^ if the study was based upon Asian population or PGC [5]. The minor allele frequency (MAF) must not have been equal to zero based upon the HapMap JPT panel. We used a Sequenom iPLEX Gold System (Sequenom, San Diego, CA) genotyping platform. In the optimization step, two SNPs (rs10193871 and rs1012053) were excluded due to a primer design problem. Moreover, because a visual inspection of the clustering revealed that six SNPs did not yield acceptable genotyping calls, we designed new primers for their proxy SNPs (N = 8) based upon tight linkage disequilibrium (LD). However, at this stage, we could not obtain optimal clustering for three of these SNPs. In total, we analyzed 45 SNPs (Figure S1 and Table S1). The quality control (QC) was conducted based upon the following criteria: (1) the missing call rate per person (less than 10%); (2) the missing call rate per SNP (less than 5%); and (3) a Hardy-Weinberg Equilibrium (HWE) P>0.0001 threshold (Table S1).

### Statistical analysis

We assessed the allelic association of the SNPs and the following three phenotypes: 1) BD (referred to as BD association); 2) SCZ (referred to as SCZ association); and 3) psychosis (BD+SCZ; referred to as psychosis association) (Figure S2 and S3).

A comparison between multiple variables is a major concern to be addressed in a genetic study in which multiple SNPs and phenotypes are analyzed. However, thus far, no gold standard has been established. Therefore, we used a two-stage analysis and stringent cut-off level for the type I error rate in the first-set screening sample. LD between SNPs selected for analysis was calculated by SNPSpD program [21], [22] to establish an effective number of independent variables (N = 36.06). We used an adjusted statistical significance level (P<0.00138) based upon this number of independent variables. The associated SNPs from the first-set screening samples were followed-up and genotyped to replicate the association in the independent second sample set. In these analyses (first-set and second-set analyses), a one-tailed analysis was applied under a unidirectional hypothesis that risk alleles identified in the original studies were associated with risk in our dataset. We assumed this association because most of the original studies that we referred to used a larger number of subjects than those in our screening datasets [11].

A meta-analysis was conducted by combining the screening, the replication and/or the original datasets. It is worth noting that if the original dataset was involved in the PGC mega-analysis of BD, we used PGC results for the meta-analysis. A fixed model (if the *I^2^* heterogeneity index was less than 50) or random effect model (if the *I^2^* heterogeneity index was greater than 50) was applied in each analysis. All of the statistical procedures were calculated using PLINK version 1.07 [23].

## Results

### Replication analysis of the BD GWAS SNPs with BD, SCZ and psychosis in the Japanese population

After the QC calculations, 42 SNPs and 2,759 samples in the first-set screening samples were eligible for the association analysis (916 patients with BD, 946 patients with SCZ and 897 healthy controls). Table 1 lists the results, which indicated a nominal association signal (uncorrected P<0.05). Complete results are presented in Table S2. It is of note that all of the SNPs that had a nominal association with the BD sample (BD association), were also associated with SCZ (SCZ association), thus the P-values for psychosis (psychosis association) are more significant (Table 1).

**Table 1:** Association analysis in the first-set screening samples (SNPs with nominal significant association).

				First-set screening										Second-set replication						
Chr	SNP	closest GENE	BP^a^	A1^b^	A2^c^	Phenotype^d^	F_A^e^	F_U^f^	P^g^	P_corrected_	OR^h^	SE^i^	direc-tion^j^	F_A	F_U	P^g^	P_corrected_	OR	SE	direction
1	rs472913	NF1A	61095558	G	C	BD	0.485	0.499	0.20	1	0.945	0.066	+							
						SCZ	0.469		**0.036**	1	0.888	0.066	+							
						Psychosis	0.477		0.063	1	0.916	0.058	+							
3	rs2251219	PBRM1	52584787	A	G	BD	0.490	0.452	0.011	0.39	1.17	0.067	+	0.485	0.460	0.042	0.085	1.11	0.059	+
						SCZ	0.500		**0.0018**	0.064	1.21	0.066	+	0.489		**0.0051**	**0.010**	1.13	0.046	+
						Psychosis	0.495		**0.0013**	**0.048**	1.19	0.058	+	0.488		**0.0035**	**0.0070**	1.12	0.042	+
3	rs1042779	ITIH1	52821011	A	G	BD	0.498	0.465	0.025	0.92	1.14	0.067	+							
						SCZ	0.503		**0.012**	0.43	1.16	0.066	+							
						Psychosis	0.500		**0.0076**	0.28	1.15	0.058	+							
3	rs736408	ITHI3	52835354	A	G	BD	0.450	0.480	0.034	1	0.885	0.067	+							
						SCZ	0.446		**0.021**	0.75	0.874	0.066	+							
						Psychosis	0.448		**0.013**	0.46	0.879	0.058	+							
7	rs10240470	MADIL1	1916497	G	T	BD	0.107	0.085	0.014	0.49	1.29	0.11	+							
						SCZ	0.098		0.090	1	1.17	0.11	+							
						Psychosis	0.102		**0.022**	0.79	1.23	0.10	+							
7	rs2709736	SP8	20862302	A	G	BD	0.305	0.345	0.0055	0.20	0.835	0.071	+							
						SCZ	0.314		**0.025**	0.88	0.871	0.070	+							
						Psychosis	0.310		**0.0046**	0.16	0.853	0.061	+							
7	rs2709722	SP8	20867808	T	C	BD	0.311	0.350	0.0063	0.23	0.838	0.071	+	0.303	0.331	0.023	0.046	0.880	0.064	+
						SCZ	0.307		**0.0024**	0.085	0.820	0.070	+	0.314		**0.049**	0.098	0.922	0.049	+
						Psychosis	0.309		**0.0010**	**0.037**	0.829	0.061	+	0.311		**0.017**	**0.033**	0.909	0.045	+
10	rs10994336	ANK3	62179812	T	C	BD	0.362	0.346	0.16	1	1.07	0.070	+							
						SCZ	0.380		**0.017**	0.62	1.16	0.069	+							
						Psychosis	0.371		**0.036**	1	1.11	0.060	+							

a: BP: base position based upon hg19.

b: A1: minor allele based upon whole sample.

c: A2: major allele.

d: BD: bipolar disorder, SCZ: schizophrenia.

e: F_A: Frequency of A1 in affected subjects.

f: F_U: Frequency of A1 in unaffected subjects.

g: P values based upon one-tailed test.

h: OR: Odds ratio for A1 (i.e. A2 is reference).

i: SE: standard error.

j: direction: direction of the effect size based upon the original studies. +: same direction. −: opposite direction.

In the analysis of BD association and psychosis association, the most significant association maps to the Sp8 transcription factor (*SP8*) locus (rs2709736: uncorrected P = 0.0055 for BD, and rs2709722, uncorrected P = 0.0010 for psychosis), which is the same direction as the original Taiwanese population-based study [16]. However, in the analysis of SCZ association, the strongest association signal maps to chromosome 3 (52–53 Mb) and rs2251219 (uncorrected P = 0.0018) in the polybromo 1 gene (*PBRM1)*.

Only two SNPs (rs2709722 in *SP8* and rs2251219 in *PBRM1*) within the psychosis set remained significant after the multiple comparison correction (corrected P = 0.037 and 0.048 for rs2709722 and rs2251219, respectively). Thus, we performed a replication analysis of these two SNPs using an independent second set of samples with BD and SCZ. The associations of both SNPs were replicated, indicating a significant association with psychosis (corrected P = 0.033 and 0.0070 for rs2709722 and rs2251219, respectively). However, the *SP8* SNP (rs2709722) and *PBRM1* SNP (rs2251219) revealed only a nominal association level with BD or SCZ (0.01<corrected P<0.1, Table 1).

### Meta-analysis of the significant SNPs detected in the first-set screening samples combining the results from our current study and the original study

In the meta-analysis, we combined our two datasets (the first-set screening samples and second-set replication samples) for the two SNPs (rs2709722 and rs2251219) to assess the association for only the Japanese population. We obtained stronger evidence of association in all of the sample sets (Table 2). Specifically, results from the psychosis sample had the most significant association (P = 8.0×10^−5^ for rs2251219 and P = 4.0×10^−4^ for rs2709722).

**Table 2:** meta analysis of the two SNPs detected in the first-set screening analysis.

					P value of original study			current study (screeing+replication)			current study+PGC BP			current study+Lee et al.			current study+Lee et al.+PGC BP		
Chr	SNP	GENE	A1^a^	A2^b^	PGC BP	Lee et al.^c^	phenotype^d^	P	OR^e^	*I^2^*	P	OR^e^	*I^2^*	P	OR^e^	*I^2^*	P	OR^e^	*I^2^*
3	rs2251219	PBRM1	A	G	5.5E-07	-	BD	0.0048	1.13	0	9.4E-09	1.13	0						
							SCZ	0.00016	1.15	0	4.3E-10	1.13	0						
							Psychosis	8.0E-05	1.14	0	2.0E-10	1.13	0						
7	rs2709722	SP8	T	C	0.089	5.06E-05	BD	0.0016	0.86	0	0.0322	0.91	56.2	5.1E-07	0.84	0	0.0070	0.88	72.9
							SCZ	0.0030	0.89	46.6	0.0332	0.92	58.4	1.6E-06	0.86	42.2	0.0074	0.89	73.9
							Psychosis	0.00040	0.88	32.4	0.0212	0.91	64.6	2.1E-07	0.86	30.3	0.0043	0.89	74.7

a: A1: first allele code.

b: A2: second allele code.

c: Lee et al. reported the P values based upon dominant model. To conduct meta-analysis of allelic model, we re-caluculated P values based upon their results.

d: BD: bipolar disorder, SCZ: schizophrenia.

e: the effect is with respect to the A1 allele.

We then combined the results from the original study [16] and/or PGC [5] datasets. For rs2251219, the original study by McMahon et al. [17] reported that the association was included in the PGC [5]; thus, we only combined the PGC results. The original study by Lee et al. [16] showed significance for rs2709722 based upon a dominant model. To conduct this meta-analysis, we recalculated the allele-wise P-value based upon the allele frequency information. For rs2251219, we detected an association with a genome-wide significance level only in the BD (P = 9.4×10^−9^), SCZ (P = 4.3×10^−10^) and psychosis sets (P = 2.0×10^−10^). Stronger evidence for rs2709722 was obtained in the meta-analysis (the current study and Lee's results [16]) (BD only: P = 5.1×10^−7^, SCZ only: P = 1.6×10^−6^, psychosis: P = 2.1×10^−7^), although this signal weakened the sample size was increased by including data from a Caucasian population (results from the current, Lee et al. and PGC studies: BD only: P = 0.0070 SCZ only: P = 0.0074 and psychosis: P = 0.0043: Table 2).

## Discussion

In this study, we conducted a two-stage association analysis of the promising risk SNPs based upon BD GWASs with BD, SCZ and psychosis samples from a Japanese population. Two SNPs were detected with significant associations in the all of the phenotypes from the first-set screening (if uncorrected for multiple comparison) and second-set replication samples, indicating that these SNPs may play a role as a common risk factor for both BD and SCZ. Furthermore, we detected an association on a genome-wide significance level within the *PBRM1* locus (rs2251219) by combining the results from the recent mega-analysis, which used the largest sample size thus far [5].

The SNP rs2251219, which maps to the *PBRM1* locus, was originally reported in a meta-analysis of BD and major depressive disorders in a Caucasian population (P = 1.7×10^−9^) [17]. This SNP was also reported in the PGC BD as possessing a suggestive level of association (P = 5.5×10^−7^) [5]. Our meta-analysis supports this finding regarding BD because we detected a genome-wide significance (P = 9.4×10^−9^), even when the BD set was analyzed alone. It is also of note that the results of this meta-analysis merging our SCZ/psychosis sets and PGC BD showed genome-wide significance (SCZ and PGC BD: P = 4.3×10^−10^, psychosis and PGC BD: P = 2.0×10^−10^).

Interestingly, rs2251219 is in LD with another SNP (rs1042779, base position  = 52.8 Mb) in inter-alpha-trypsin inhibitor heavy chain 1 (*ITIH1*), which was examined in our screening sample (D' = 0.96 and r^2^ = 0.84 in an Asian population in the 1000 genome database as a reference panel) and revealed a nominal association (uncorrected P<0.05 for all phenotypes). This region (3p21.1) is one of the most attractive loci as a candidate region for risk for psychotic disorders. A recent study by Hamshere et al. [11] reported that SNP (rs2239547) in inter-alpha-trypsin inhibitor heavy chain 3 and 4 (*ITIH3-4*) was significantly associated with BD and/or SCZ at a genome-wide significance level (D' = 0.95 and r^2^ = 0.58 with rs2251219, Asian population in the 1000-genome database as a reference panel). The most recent study by the PGC Cross-Disorder Group (CDG) also reported that SNP (rs2535629) in *ITIH3-4* was associated with five psychiatric disorders, and the strongest association was observed in the BD set [12]. These SNPs located in and around *PBRM1*, *ITIH1* and *ITIH3-4* are in strong LD. Thus, all of the SNPs in this LD block are promising candidates for genetic risk for BD, SCZ or psychosis. This LD structure in turn indicates that it is difficult to narrow down the true susceptibility variants within this region (Figure S4).

A Taiwanese BD GWAS found a suggestive association signal that maps to the *SP8* locus (P = 4.8×10^−7^ in dominant model) [16]. Our result supports the association of this gene with BD, specifically for Asian populations. The meta-analysis of our results and Lee's results indicated a stronger association signal that was weakened when the sample size was increased by including data from a Caucasian population (Table 2). Considering the results of the heterogeneity test (in which the *I^2^* score significantly increased by combining PGC BD dataset), the *SP8* gene may play a role as a population-specific risk gene in individuals of Asian ancestry. *SP8* is a SP transcription factor and functions in neural development by interacting with Wnt/beta-catenin and fibroblast growth factor (FGF) signaling [24]. Because there are no studies that have examined the association between *SP8* and BD/SCZ, further research is needed to better understand the relationship between *SP8* and psychiatric disorders. Special attention is needed regarding the population-specific effect that rs2709722 might have.

SNPs located in other promising candidate genes were not significantly associated with BD, SCZ or psychosis in the Japanese population. Although a trans-population effect is a likely explanation, our sample size may not have sufficient power to observe the association compared with the estimated effect size (odds ratio (OR) of ∼1.2). The analysis of the power of our study [25] indicated that our sample had 25% power for BD/SCZ and 40% for psychosis to detect significance (assuming an OR of 1.2) of risk with 25% MAF (average MAF in our examined SNPs in the control subjects) under an additive model (type I error rate = 0.00138). Therefore, a larger sample size in future work is essential.

In conclusion, we found two loci, *PBRM1* (and neighboring genes) and *SP8*, that were replicated in psychotic disorders in a Japanese population. Specifically, a SNP within *PBRM1* revealed genome-wide significance in the meta-analysis, suggesting promising candidate genes for BD. *SP8*, which was not significant on a genome-wide level, is still a candidate for a population-specific risk factor for BD.

## Supporting Information

Figure S1 **SNP selection strategy.** JPT: HapMap Japanese Tokyo sample HWE: Hardy-Weinberg Equilibrium.
(TIF)

Figure S2 **Sample numbers in the first-set screening samples.** BD: Bipolar disorder SCZ: Schizophrenia.
(TIF)

Figure S3 **Sample numbers in the second-set replication samples.** BD: Bipolar disorder SCZ: Schizophrenia.
(TIF)

Figure S4 **Linkage disequilibrium structure around 3q21 in the Asian population.** Data are from HapMap JPT and CHB. The LD measure is based upon D'.
(TIF)

Table S1 **SNPs selected based upon previous BD GWAS.**
(XLSX)

Table S2 **Association analysis for all SNPs.**
(XLSX)
