# Supplementary figures and images for: Genetic Variants on 3q21 and in the Sp8 Transcription Factor Gene (SP8) as Susceptibility Loci for Psychotic Disorders: A Genetic Association Study
Source: PLoS One. 2013 Aug 13;8(8):e70964. doi: 10.1371/journal.pone.0070964 (PMC3742587; doi:10.1371/journal.pone.0070964)

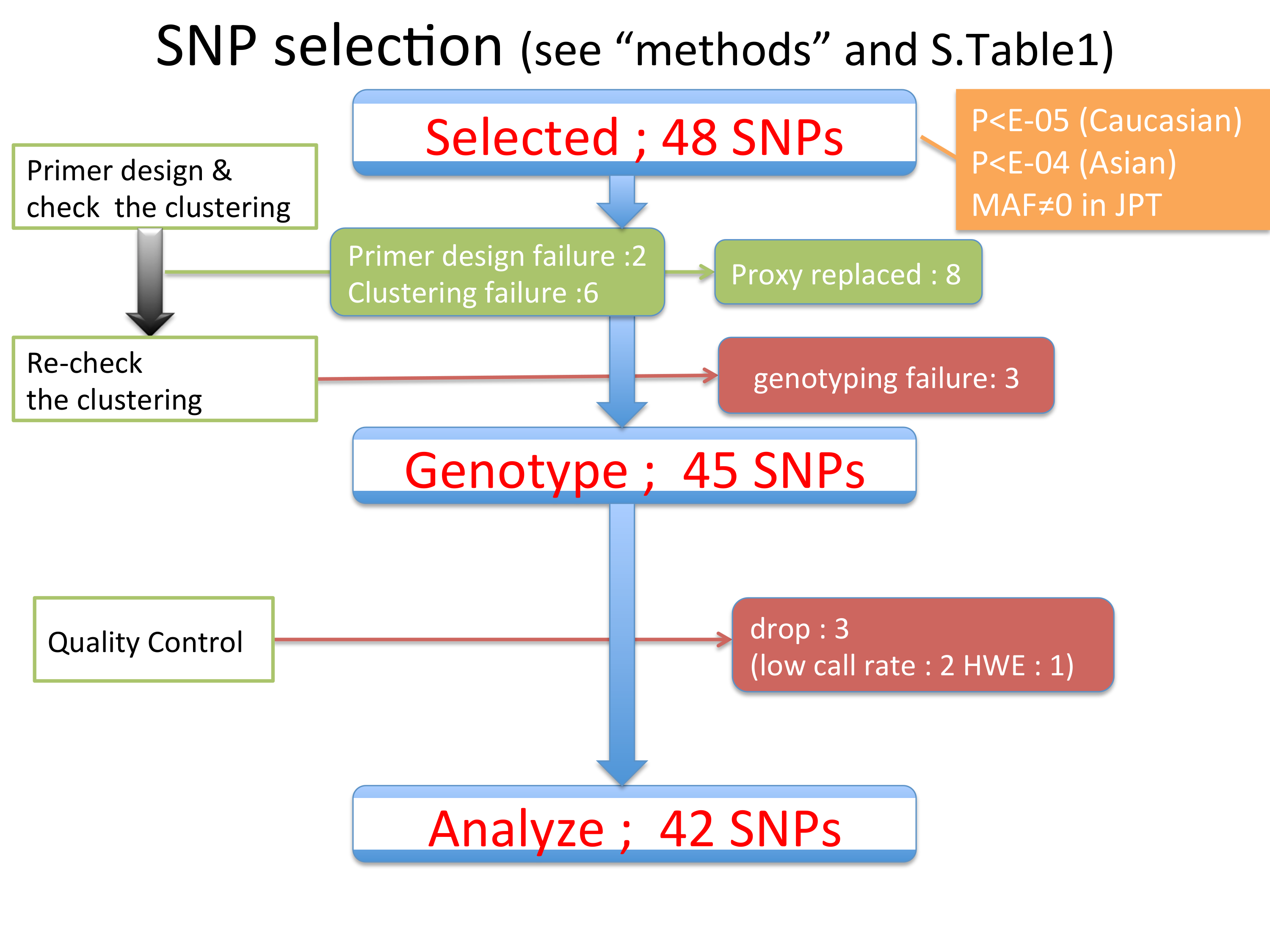

Supplement: Figure S1 — SNP selection strategy. JPT: HapMap Japanese Tokyo sample HWE: Hardy-Weinberg Equilibrium. (TIF) [file pone.0070964.s001.tif]

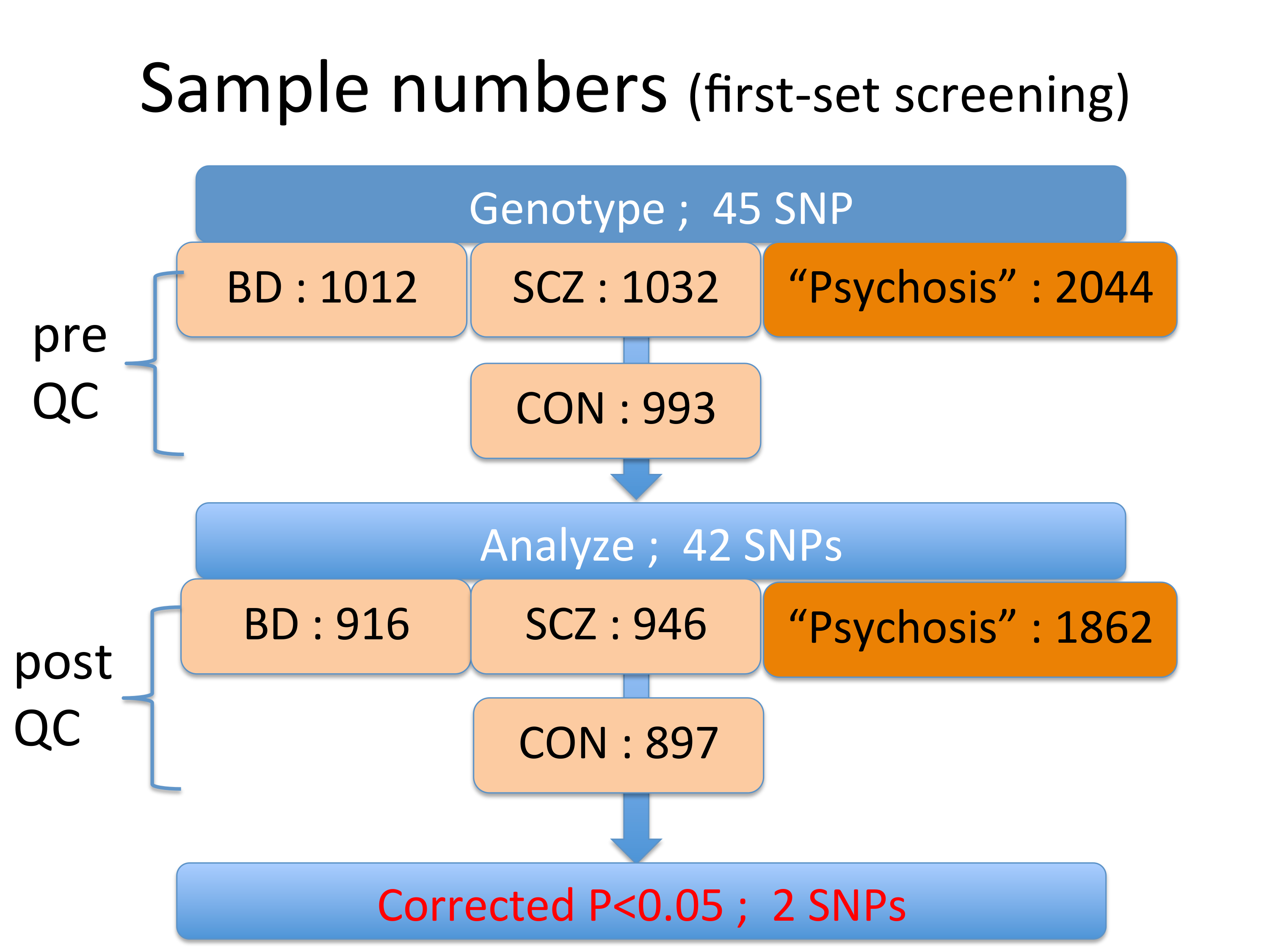

Supplement: Figure S2 — Sample numbers in the first-set screening samples. BD: Bipolar disorder SCZ: Schizophrenia. (TIF) [file pone.0070964.s002.tif]

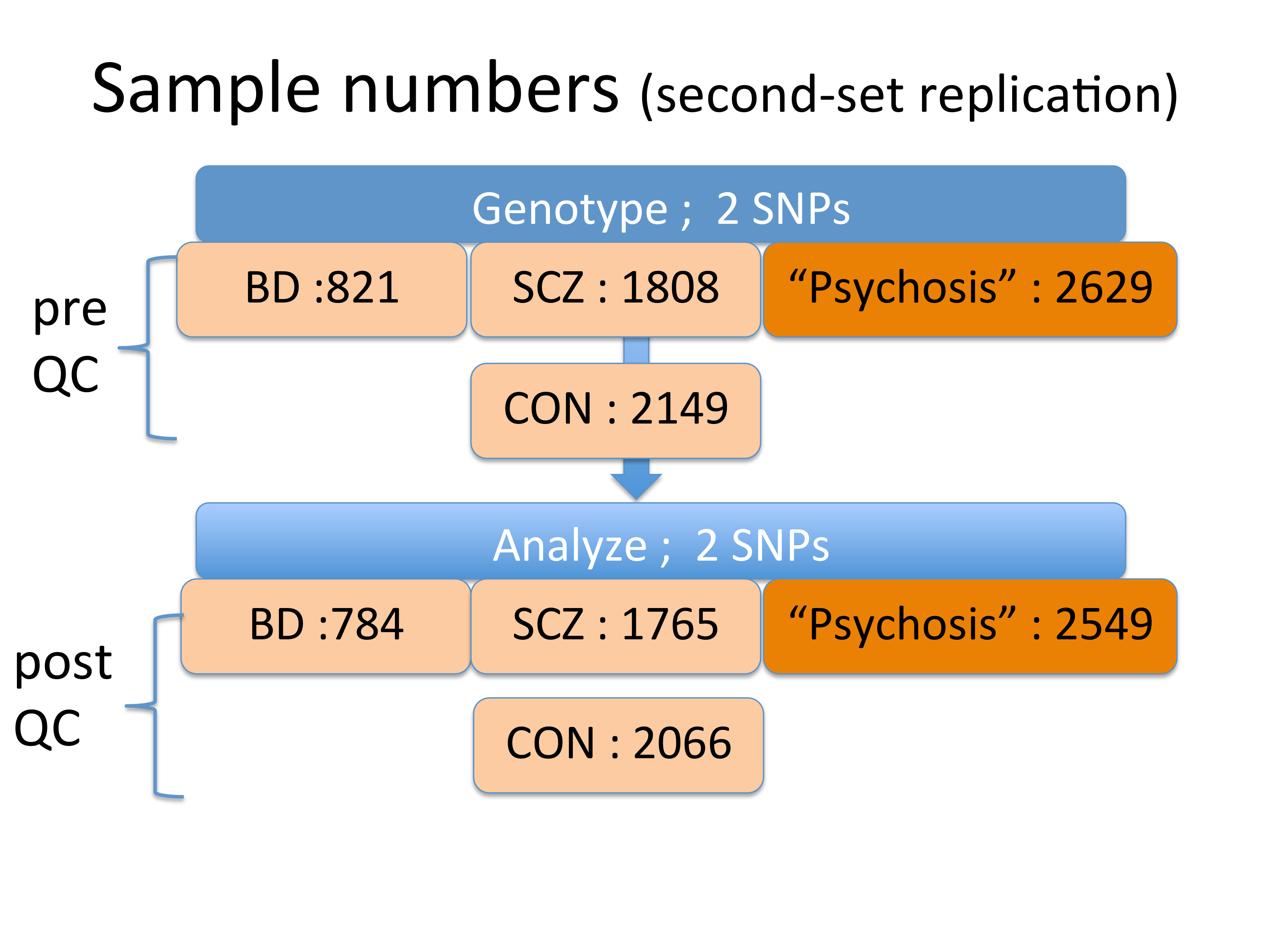

Supplement: Figure S3 — Sample numbers in the second-set replication samples. BD: Bipolar disorder SCZ: Schizophrenia. (TIF) [file pone.0070964.s003.tif]

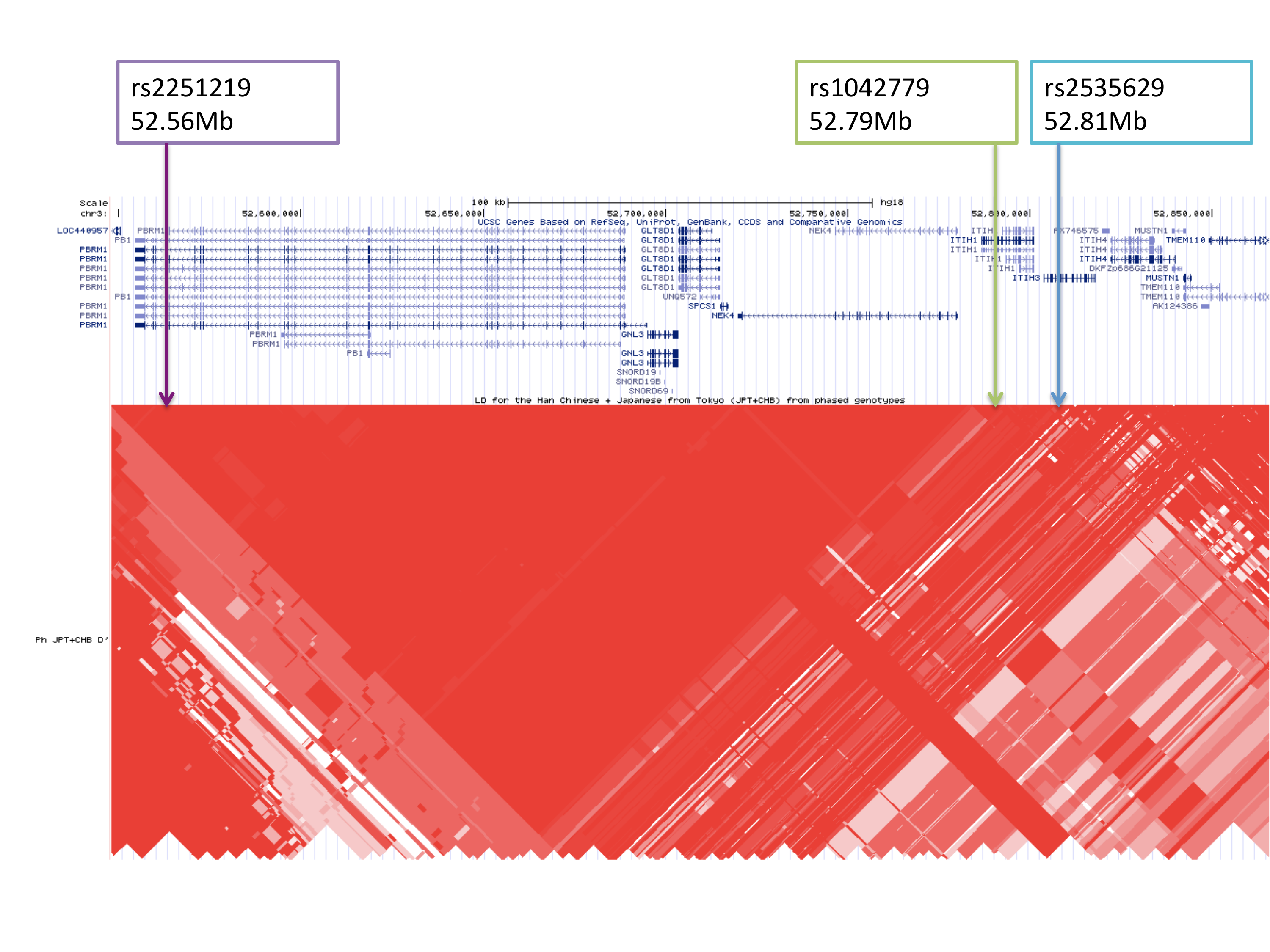

Supplement: Figure S4 — Linkage disequilibrium structure around 3q21 in the Asian population. Data are from HapMap JPT and CHB. The LD measure is based upon D'. (TIF) [file pone.0070964.s004.tif]
